# Validation of reference genes for quantitative real-time PCR in tiger beetles across sexes, body parts, sexual maturity and immune challenge

**DOI:** 10.1038/s41598-018-28978-7

**Published:** 2018-07-16

**Authors:** Andrés García-Reina, María Juliana Rodríguez-García, José Galián

**Affiliations:** University of Murcia, Department of Zoology and Physical Anthropology, Faculty of Veterinary, Campus Mare Nostrum, E-30100 Murcia, Spain

## Abstract

Reference genes are frequently used as normalizers for expression studies despite not being previously verified to present suitable stabilities. Considering the interest that tiger beetles have generated in the past years, resulting in a variety of studies, it is crucial to dispose of a validated reference gene panel for expression studies. Nine candidate genes were tested in *Cicindela campestris* and *Calomera littoralis* across several conditions and their transcription levels were assessed with *geNorm*, *NormFinder*, *BestKeeper* and *ΔC*_*T*_ *method* algorithms. Results showed high stabilities across sexes, immune challenge and gonad developmental stages for all genes tested, while body parts comparison presented less constant expression values. Only two genes are sufficient to perform a proper normalization for most of the conditions tested, except for the body parts comparison in *C*. *littoralis*, which requires the use of at least three reference genes. On the whole, no universal gene is found to be suitable for all situations, but according to the acceptable range of values, *NADH*, *B-t*, *Vatpase* and *ArgKin* seem to present the most constant expression stability, indicating their suitability as reference genes in most of the conditions. This is the first report evaluating the stability of housekeeping genes in adephagan beetles.

## Introduction

Evaluating changes in gene expression has lately been one of the most frequent studies to analyse the effect of physiological responses and therefore the biological differences among populations. Quantitative real-time polymerase chain reaction (RT-qPCR) has recently become the most widely accepted methodology which accurately and with high reproducibility generates expression profiles and allows their interpretation^1–4^. Nevertheless, some guidelines must be followed to correctly perform a reliable RT-qPCR analysis^5–8^, being essential the use of an internal control so that any other variation across samples derived from handling or cDNA synthesis can be avoided^3,7–11^. The normalization is then carried out by introducing reference genes known to present a constant expression profile after alterations in a specific condition. Many housekeeping genes (HKG) have been incorrectly described and used as reference genes without previously testing their viability due to the assumption that their role in basic cellular processes grants them a complete and suitable stability. However, it has been established that most of these genes can alter their expression under certain conditions^12–16^, thus it is crucial to perform a proper validation to determine the suitability of any candidate reference gene for each specific condition.

Several studies validating reference genes have been carried out in the last years, some of them involving insect species. Validation in *Bemisia tabaci*^17^ selected genes for biotic and abiotic conditions. Ponton *et al*.^18^ checked the suitability of references genes in *Drosophila melanogaster* after an injury, heat stress and different diets, and Koramutla *et al*.^19^ recommended several reference genes in *Lipaphis erysimi*. Additionally, gene stability was tested in different conditions in two *Bombus* species^20^, while Lourenço *et al*.^21^ studied the suitability in *Apis mellifera*. More specifically, reference genes validation has also been carried out in polyphagan beetles. That is the case of *Tribolium castaneum*^22–24^, for which different ribosomal proteins were found to be the suitable for broad-spectrum expression analyses. Two different species of Coccinellidae^25,26^, the western corn root worm *Diabrotica virgifera virgifera*^27^ and the emerald ash borer *Agrilus planipennis*^28^ have also been investigated.

Cicindelids, commonly known as tiger beetles, are one of the most important non-pest species within the suborder Adephaga (Coleoptera), with more than 2500 species described^29^. They are worldwide distributed with the exception of Tasmania, Antarctica and some remote Oceanic islands, occupying a wide range of habitats^30,31^, and are considered to be good bioindicators due to their biological and evolutionary characteristics^30,32–34^. Moreover, the genomic study of non-model species, especially with the modern technologies currently available, provides important information to understand the genetic basis of adaptive traits^35^. Nevertheless, only a few studies have been performed in cicindelids regarding the study of their transcriptome, in relation to their reproduction and innate immune response^36–38^ but there is no information available concerning the stability of HKGs in this group of beetles. The availability of a selection of validated reference genes for genomic and expression analyses will allow us to obtain more reliable results in future studies.

Nine candidate reference genes were evaluated across different conditions in *Cicindela campestris* and *Calomera littoralis* in this study, including Arginine Kinase (*ArgKin*), Myosin regulatory light chain 2 (*Myo*), Myosin light chain alkali (*Myo-Alc*), 60 s acidic ribosomal p0 (*60Sa*), Ribosomal protein S18 (*RPS18*), Tubulin beta-1 chain (*B-t*), Vatype proton atpase 16kda proteolipid subunit (*Vatpase*), NADH dehydrogenase (*NADH*) and 40 s ribosomal protein s11 (*RPS11*). The aims of this research are (i) to examine the expression stability of different housekeeping genes in non-model beetle species across sexes, body parts, gonad developmental stages (immature/mature) and immune challenge (ii) to provide a selection of accurate reference genes for future genomic and expression analyses in cicindelids.

## Results

### Selection of reference genes

Nine genes were identified as putative reference genes according to blast searches from both libraries: (i) Arginine Kinase (*ArgKin*), Myosin regulatory light chain 2 (*Myo*), Myosin light chain alkali (*Myo-Alc*) and 60 s acidic ribosomal p0 (*60Sa*) were obtained from *C*. *littoralis*; (ii) Ribosomal protein S18 (*RPS18*), Tubulin beta-1 chain (*B-t*), Vatype proton atpase 16kda proteolipid subunit (*Vatpase*), NADH dehydrogenase (*NADH*) and 40 s ribosomal protein s11 (*RPS11*) were identified from *C*. *campestris*.

**Figure 1:**
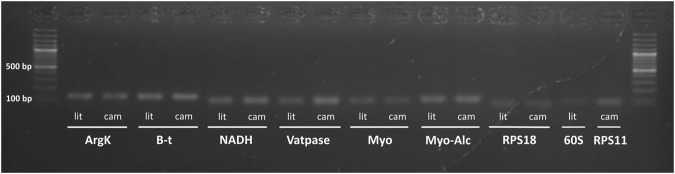
Agarose gel showing the fragments amplified with all the primers selected for qPCR analyses. All fragments showed predicted lenghts for both species (lit: *C*. *littoralis*; cam: *C*. *campestris*). Gel is presented uncropped and no posterior image edition and modification in exposure or contrast were made.

**Figure 2:**
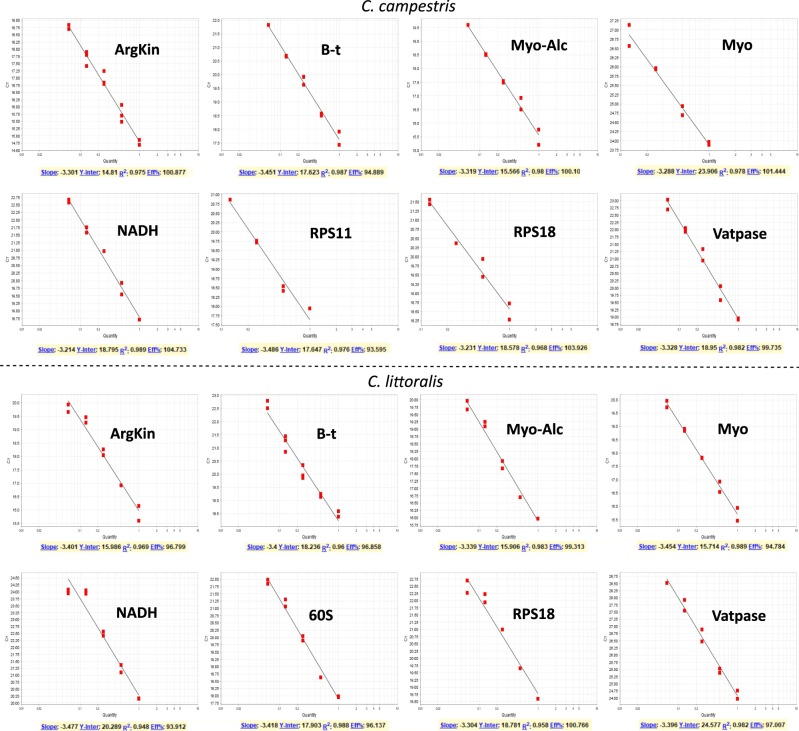
Standard Curves of all genes tested for both of the species studied showing efficiencies and the slope and R^2^ values obtained.

**Figure 3:**
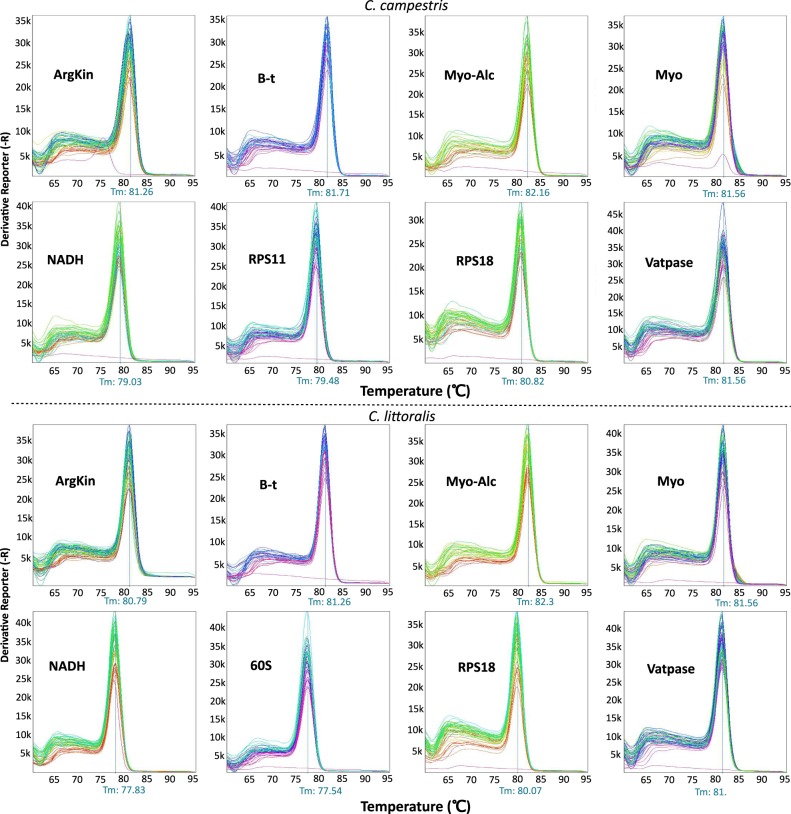
Melt curves for each gene and species studied. Each graph represents all the curves for all conditions tested for that gene. Peaks indicate the melt temperature for each fragment amplified, showing single peaks with no dimer primer or contamination peak observed.

All genes amplified a single fragment with the predicted length (Fig. 1), and were tested for amplification efficiencies in both species to check their stability in expression analyses. Efficiencies ranged between 93.9% and 104.8% in both species (Fig. 2; Table 1), except for RPS11 in *C*. *littoralis* and 60Sa in *C*. *campestris* which did not amplify correctly with efficiencies outside the acceptable range to be considered as viable for RT-qPCR analyses, and thereby they were discarded for each corresponding species. Melt curves showed the expected single peaks indicating the specificity of the amplifications (Fig. 3).

**Table 1:** Primers used for qRT-PCR experiments for both *C*. *campestris* (cam) and *C*. *littoralis* (lit). E = amplification efficiencies.

Gene	Full gene name	Species amplified	E (%) *C*. *campestris*	E (%) *C*. *littoralis*	Amplicon length (bp)	Primers
Argkin	Arginine Kinase	cam/lit	101,9	95,58	130	F-5′-CTCGTGTGGTGCAACGAAGA-3′ R-5′-GGTGGCTGAACGGGACTCT-3′
RPS18	Ribosomal protein S18	cam/lit	103,9	100,7	80	F-5′-TTCGTGCCCATCGTGGTAT-3′ R-5′-GCCGCGTCGTCCAGTAGTT-3′
B-t	Tubulin beta- 1 chain	cam/lit	94,8	96,8	130	F-5′-GAGGTGGACGAGCAAATGCT-3′ R-5′-TGAAGGTGGCGGACATTTTC-3′
Vatpase	Vatype proton atpase 16kda proteolipid subunit	cam/lit	99,7	97	100	F-5′-GACGAGGTTGTCGTGCTGTTC-3′ R-5′-GGTGCTGGATTAGCTGTTGGA-3′
Myo	Myosin regulatory light chain 2	cam/lit	100,1	94,7	100	F-5′-CACGACAAAGACGGCATCAT-3′ R-5′-CGTTCAGCATCTCGTCGAGTT-3′
NADH	NADH dehydrogenase	cam/lit	104,7	93,9	100	F-5′-AGGCGGCTCCATCACCTAA-3′ R-5′-GTCGACGGAAACGCTAAACC-3′
Myo-Alc	Myosin light chain alkali	cam/lit	101,4	99,3	110	F-5′-CAAGGGTTGGGTTCAGATTCA-3′ R-5′-TGAGAGGGCGAACTTCGTCTT-3′
RPS11	40 s ribosomal protein s11	cam	101,9	—	100	F-5′-GGTTCGAAAAACGTCACCGTAA-3′ R-5′-AAGGGCCGACATTCTCCAAT-3′
60Sa	60 s acidic ribosomal p0	lit	—	96,1	100	F-5′-GCTCCAAGCAAATGCAACAA-3′ R-5′-AGGTGGCCTCGGATAGCTTT-3′

### Stability of the candidate reference genes

Eight candidate reference genes in each species were analysed by RT-qPCR to validate their stability and therefore their suitability for expression analyses under different conditions. C_T_ values obtained were used as input data for stability analysis with all the four tools. *geNorm* and *NormFinder* uses Ct values transformed to logarithmic scale adjusted to the minimum value for each condition, while *Bestkeeper* and *ΔC*_*T*_ *method* use raw data. All results and stability ranks are shown in Table 2 and Table 3.

**Table 2:** Ranking of candidate reference genes based on stability values obtained with *geNorm*, *NormFinder*, *Bestkeeper* and *ΔC*_*T*_ methods in *C*. *campestris*.

Condition	Reference gene	GeNorm		NormFinder		BestKeeper		ΔC_T_	
		Stability	Rank	Stability	Rank	Stability	Rank	Stability	Rank
Sex	ArgKin	0,718	7	0,407	5	0,58	3	0,81	6
	RPS18	0,507	4	0,431	7	0,98	8	0,80	5
	B-t	0,477	1	0,109	1	0,63	4	0,58	1
	Vatpase	0,544	5	0,349	2	0,50	1	0,69	2
	Myo	0,983	8	0,541	8	0,51	2	0,99	8
	NADH	0,477	1	0,404	4	0,86	5	0,76	4
	Myo-Alc	0,481	3	0,354	3	0,91	6	0,70	3
	RPS11	0,573	6	0,415	6	0,96	7	0,82	7
Gonads development	ArgKin	0,469	5	0,312	5	0,65	7	0,63	4
	RPS18	0,343	1	0,163	1	0,46	4	0,50	1
	B-t	0,470	5	0,295	4	0,59	5	0,63	4
	Vatpase	0,378	4	0,333	7	0,35	3	0,71	7
	Myo	0,496	7	0,325	6	0,65	7	0,70	6
	NADH	0,343	1	0,181	2	0,33	2	0,56	2
	Myo-Alc	0,619	8	0,344	8	0,64	6	0,73	8
	RPS11	0,374	3	0,258	3	0,23	1	0,56	2
Body parts	ArgKin	1,350	6	0,647	5	0,68	3	1,27	5
	RPS18	1,150	3	0,584	3	0,85	5	1,18	4
	B-t	1,306	5	0,689	6	0,96	6	1,28	6
	Vatpase	1,052	1	0,552	2	0,62	2	1,08	2
	Myo	1,871	7	0,899	7	1,31	7	1,66	7
	NADH	1,183	4	0,531	1	0,74	4	1,11	3
	Myo-Alc	2,069	8	1,028	8	1,42	8	1,80	8
	RPS11	1,058	2	0,585	4	0,59	1	1,07	1

**Table 3:** Ranking of candidate reference genes based on stability values obtained with *geNorm*, *NormFinder*, *Bestkeeper* and *ΔC*_*T*_ methods in *C*. *littoralis*.

Condition	Reference gene	GeNorm		NormFinder		BestKeeper		ΔC_T_	
		Stability	Rank	Stability	Rank	Stability	Rank	Stability	Rank
Sex	ArgKin	0,305	4	0,205	3	0,69	1	0,49	3
	RPS18	0,341	5	0,247	5	0,78	6	0,54	5
	B-t	0,259	1	0,222	4	0,76	3	0,50	4
	Vatpase	0,417	6	0,271	6	0,98	8	0,61	8
	Myo	0,268	3	0,197	2	0,76	3	0,47	2
	NADH	0,465	7	0,278	8	0,76	3	0,59	7
	Myo-Alc	0,259	1	0,144	1	0,69	1	0,41	1
	60Sa	0,487	8	0,271	6	0,93	7	0,55	6
Infection	ArgKin	0,374	4	0,231	3	0,57	2	0,61	3
	RPS18	0,752	8	0,386	8	0,68	6	0,84	8
	B-t	0,319	3	0,244	4	0,74	7	0,65	5
	Vatpase	0,438	5	0,335	6	0,32	1	0,77	6
	Myo	0,315	1	0,181	2	0,63	4	0,57	1
	NADH	0,315	1	0,176	1	0,58	3	0,58	2
	Myo-Alc	0,540	7	0,364	7	0,95	8	0,81	7
	60Sa	0,494	6	0,293	5	0,67	5	0,62	4
Body parts	ArgKin	1,447	3	0,542	3	2,08	6	1,31	7
	RPS18	1,669	6	0,822	5	1,29	3	0,90	3
	B-t	1,648	5	0,835	6	1,07	1	0,93	4
	Vatpase	1,438	2	0,641	4	1,62	4	1,36	8
	Myo	1,784	7	0,845	7	2,49	7	0,88	2
	NADH	1,261	1	0,300	1	1,68	5	1,20	6
	Myo-Alc	2,092	8	1,057	8	2,73	8	1,03	5
	60Sa	1,457	4	0,531	2	1,08	2	0,79	1

### geNorm

**Figure 4:**
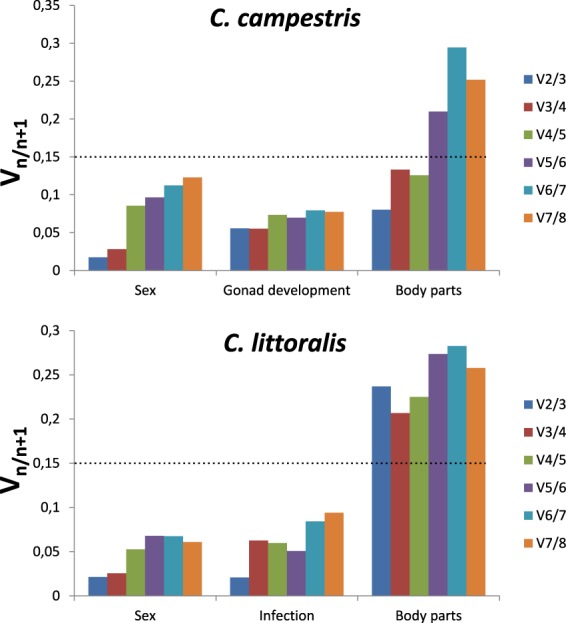
Optimal number of reference genes for normalization in *Cicindela campestris* and *Calomera littoralis*. Pairwise variation V_n/n+1_ values of candidate reference genes for the different conditions tested in both tiger beetles species were calculated with *geNorm* software by analysing the parwise variation (V) between the normalization factors NF_N_ and NF_N+1_. Values < 0.15 are indicating that additional genes are not necessary for normalization.

Two different values are obtained from *geNorm* analyses. The average expression stability (M) establishes a ranking of genes based on those with the lowest values, considered as the most stable genes for each specific condition. On the other hand, the pairwise variation (V) provides information on the number of genes needed to perform an accurate normalization in expression analyses, and values should be below 0.15 to accept their reliability (Fig. 4).

**Figure 5:**
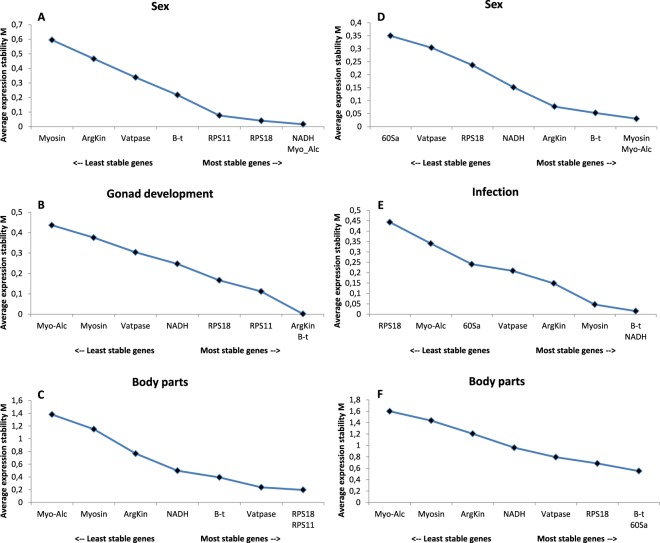
Average stability values (M) of remaining reference genes during stepwise exclusion for *Cicindela campestris* (**A–C**) and *Calomera littoralis* (**D–F**). Analysis was performed by *geNorm* for all conditions tested.

In *C*. *campestris*, the highest ranked genes for sex differences were *B-t* and *NADH*, and *Myo* was the gene with the lowest stability. However, all studied genes present values below 1 and their stability was very similar for all of them. Results are similar for the gonad development experiment, where *NADH* and *RPS18* are the most stable genes and *Myo-Alc* the gene with the highest value. Again, all genes showed values below 1. Alternatively, body parts comparison generated values above 1, with *Vatpase* as the most stable gene and Myo and Myo-Alc presenting values over 1.5, being considered the upper limit for which a gene is not behaving in a stable way. Additionally, Fig. 5 show the stability values of the remaining reference genes at each step after a stepwise exclusion analysis, which showed slightly different results.

*C*. *littoralis* differences between sexes showed high stability among all genes tested (M < 1), especially in B-t and *Myo-Alc*, similar to those values obtained for the infection treatments, being *Myo* and *NADH* the genes with the most constant expression. Nevertheless, four of the genes tested were located outside the acceptable values of M for the body parts comparison, with only *ArgKin*, *Vatpase*, *60Sa* and *NADH* below 1.5 values.

Regarding pairwise variation analyses, results showed that only two genes were necessary for most of the conditions studied, with the exception of the body parts comparison in *C*. *littoralis*, where the analysis is unable to provide an optimal number of genes to normalize the expression of target genes, even when six or seven genes were included in the estimations.

### Normfinder

Those genes with the lowest stability values obtained with *NormFinder* algorithm, based on intra- and inter-group expression variations, are considered to be the most stable reference genes.

Results in *C*. *campestris* were equivalent to those generated by *geNorm*. *B-t* is established as the most consistent gene and *Myo* presented the highest stability value for the sexes comparison. Similarly, *RPS18* had the lowest value for the gonad development experiment and *Myo-Alc* was the less stable. Both experiments generated proper stability values for all genes tested. Results for body parts comparison slightly differ from *geNorm* output. While *Myo-Alc* was again located as the less constant gene, *NADH* showed the highest stability, with *Vatpase* in second place.

All stability values obtained between sexes and infection experiment in *C*. *littoralis* were below 0.3 and close to each other, with *Myo-Alc* and *NADH* as the most stables genes, respectively. *NormFinder* considered *60Sa* to be the best reference gene for body parts comparisons, even though values were generally higher than previously reported.

### Bestkeeper

The *Bestkeeper* algorithm ranks the candidate references genes by calculating a std dev [±CP] which should not exceed 1 to validate the stability of a gene.

In *C*. *campestris*, *Vatpase* showed the lowest value when comparing between male and female individuals. Similarly, all genes presented values below 1 for the gonads development comparison, where *RPS11* was the most stable gene. *RPS11* was also considered as the most stable gene for the body parts comparison.

*ArgKin* and *Myo* share the position as the most consistent genes for the sex comparison in *C*. *littoralis*, and *Vatpase* led the reference genes in the infection experiment. While all genes in those two experiments presented values below 1, the opposite occurs when analysing values for the body parts comparison, where all genes showed values above 1. B-t and 60Sa displayed the closest values to 1 and therefore the ones with a higher stability.

### *ΔC*_*T*_ method

**Figure 6:**
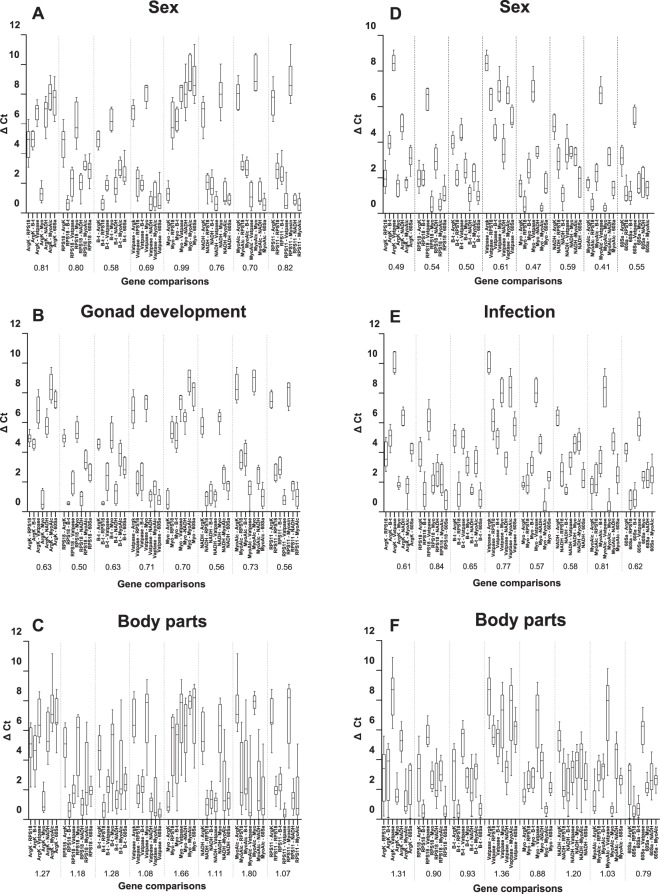
*ΔC*_*T*_ approach to reference gene selection in *Cicindela campestris* (**A–C**) and *Calomera littoalis* (**D**–**F**) for each condition tested. ΔC_T_ values are indicated as medians (horizontal lines), 25^th^ to 75^th^ percentile (boxes) and ranges (whiskers). Values in x-axis indicate the means of the standard deviations for each gene comparison. The lowest values correspond to the highest stabilities.

This method uses the standard deviation means of the C_T_ differences between each gene and the rest of them within each condition (Fig. 6). A standard deviation below 1 indicates an appropriate stability.

Both sex experiment and gonad development in *C*. *campestris* presented all genes with values below 1, but B-t and *Myo-Alc* were the genes with lower stdv mean, respectively. Contrarily to the previous methods, none of the genes were stable enough for a body part comparison according to *ΔC*_*T*_ *method*. But, for all that, *RPS11* with a value of 1.07 and *Vatpase* with 1.08 were the closest to 1 and therefore the most stables genes.

For *C*. *littoralis* the most proper gene for a sex comparison was *Myo-Alc*, while *Myo* obtained the lowest value for infection analyses. None of the genes in both analyses showed values above 1. In this case, body parts comparison identified several stable genes, being *60Sa* the one with a lower value.

By and large, all four methodologies showed similar results, with slightly variation in some gene positions, especially for the body parts comparison experiment, which took into account four different variables and generated some differences among methods and lower stabilities, mainly those obtained with *Bestkeeper* and *ΔC*_*T*_ *method*

### qPCR experiment

**Figure 7:**
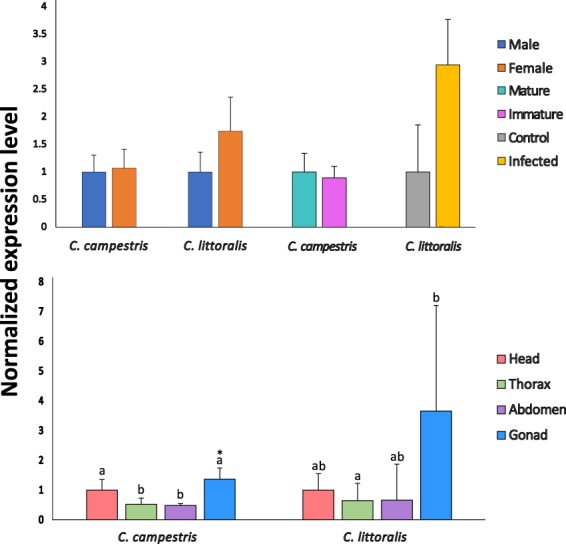
Relative expression of Clit-Def gene (control and infected samples) and UBC9 gene (rest of the samples) using the reference genes selected for each condition in both species of tiger beetles. Male, mature, control and head samples are used as normalizer for each experiment. Error bars represent the Standard Error for the Normalized Relative Quantity. Letters are indicating significant differences with a p value < 0.05, while asterisks indicate p values < 0.01.

An ubiquitin and defensin genes were tested to check their expression using as reference genes those previously validated in this work (Fig. 7). Changes in expression were observed among different conditions, specially for the body parts comparison, where gonads showed a higher ubiquitin expression than other tissues as thorax or abdomen. For the case of the defensin gene, an over-regulation of almost 3 fold-change in its expression was observed after infection, but no significance was found after the statistical analysis. Sex comparison and gonad development presented a less ubiquitin regulation than other conditions.

## Discussion

Performing a proper reference gene validation for RT-qPCR experiments is crucial for a correct reliability of the results. Thus, stability of reference genes must be proved for each condition and species used in an experiment to assure an accurate data interpretation^7,14^.

In this study, nine candidate reference genes are tested for the first time in the tiger beetles species *C*. *campestris* and *C*. *littoralis* across various conditions. Most stable genes are identified to be used in RT-qPCR for comparisons across sexes, gonads development stages, infection and body parts, using four statistical methods such as *geNorm*, *NormFinder*, *Bestkeeper* and *ΔC*_*T*_ *method*^39–42^. There are several studies regarding the validation of reference genes in other insects, but no information has previously been reported in any adephagan beetle species.

All genes tested for the sex comparison in both species showed low stability values to consider them suitable to be used as reference genes. Nevertheless, *B-t* presented the best values in *C*. *campestris* for this condition. Even when the rest of conditions never showed this gene as the most constant it is still inside the acceptable range of stability. *B-t* is one of the major components of microtubules which function in many cellular processes. Previous works in insects have shown its suitability for developmental stages, tissues and dietary RNAi experiments in *Coccinella septempunctata*^26^, developmental stages in *Liposcelis Bostrychophila*^43^ and across all the conditions tested in *Drosophila melanogaster*^18^, while it has also been tested in *Tribolium castaneum*^24^, *Bemisia tabaci*^17^, *Dibrotica virgifera virgifera*^27^, *Apis mellifera*^44^ and two *Bombus* species^20^, not being ranked as the optimal gene in most of the cases. Even though *Myo* and *Myo-Alc*, related to ATP regulation and cell movement, were allocated as the best genes for sex comparison in *C*. *littoralis*, they were proved to be the least recommended genes for most of the conditions tested. That is equivalent to the results obtained by Li *et al*.^17^ for biotic and abiotic conditions in *Bemisia tabacii*. *Myo* was also considered as a putative reference gene after a viral infection in *Spodoptera frugiperda* cells^45^ which is in agreement with the results obtained in this study, but it was discarded due to its high C_T_ values.

Some of the most used genes in identification of reference genes studies are the ribosomal proteins, which are involved in the cellular process of translation. Three of them have been tested in this work but only *RPS18* was used for both species because *RPS11* primer efficiencies in *C*. *littoralis* were not good enough to allow its use in expression studies, and exactly the opposite case occurred for *60Sa*. Their best stability is observed in the gonads development comparison in *C*. *campestris* as both *RPS18* and *RPS11* were top-ranked by all four algorithms. *RPS11* was ranked among the most stables genes for the body parts comparison in *C*. *campestris*, contrary to *RPS18* which was not placed among the top-ranked genes in either both species. Previous studies in insects showed good stabilities of ribosomal proteins in developmental and tissue comparisons^23,28,46–48^. Nevertheless, *RPS18*, which was the most constant gene after bacterial infection in *Apis mellifera*^44^, showed the least stable values in *C*. *littoralis* after the treatment. This highlights the necessity of a proper validation to find the optimal reference gene for each species and specific condition. Additionally, several ribosomal proteins were also validated in *T*. *castaneum* after a fungal challenge^22^, RPS9 was proved to be suitable for RNAi experiments^27^, and *RPS18* and *RPL13* were selected among the best reference genes in the mustard aphid *Lipaphis erysimi*^19^. Lastly, 60Sa was only used for *C*. *littoralis* conditions and its best stability value is shown for body parts comparison.

*NADH* plays a key role in cellular respiration and was selected as one of the most stable genes across all conditions tested by most of the methods used. It is one of the best genes together with the ribosomal proteins for gonad development comparison and was considered by g*eNorm* and *NormFinder* as the most constant gene in *C*. *littoralis* after the infection and for the body parts comparison. Despite of not being the top-ranked gene in the other conditions tested, it still presents good stability values within the acceptable range. Li *et al*.^17^ verified its usefulness in *Bemisia tabaci* for abiotic factor, but it also presented good values for some biotic factor such as developmental stages. In addition, NADH was top-ranked as one of the most stables gene for developmental stages and tissue comparison in *Coccinella septempunctata*^26^ and exhibited acceptable values in *Coleomegilla maculata*^25^.

*ArgKin*, known to function as an energy and ATP regulator, has not been a common reference gene in previous studies, some of them not presenting it as a top-ranked gene for expression analyses in insects^25,26,47^. Alternatively, it was found to be suitable for studies in two *Bombus* species by Horňáková *et al*.^20^. In this study, it was neither ranked as the most stable gene for any of the conditions tested nor among the worst. It displayed a constant stability across all treatments with most of its values within an acceptable stability range, which may be indicating that, not being the best gene for any condition tested, it could be used as reference genes in several situations along with other validated genes. A similar situation occurs in the case of *Vatpase*, protein that acts as a proton pumb in cell membranes. Its best stability is displayed for body parts and sex comparisons in *C*. *campestris* but most of the time is ranked in the middle of the gene distribution. Previous studies in *Bicyclus anynana*^49^, *Coleomegilla maculate*^25^ *and Coccinella septempunctata*^26^ support these results, as *Vatpase* was never positioned among the most constant genes.

Stability values for both species body parts comparisons were considerably higher than those obtained for the rest of conditions. Moreover, no genes were obtained which presented acceptable values according to *ΔC*_*T*_ *method* in *C*. *campestris*, and that was also the case for *C*. *littoralis* with *BestKeeper* results. That is likely due to the complexity of all body parts and tissue, which were divided into head, thorax, abdomen and gonads, each of them composed by different tissues which could be generating a higher variability in the expression levels of all genes.

Regarding the optimal number of genes for normalization in expression experiments, the results from the pair-wise variation of the normalization factors (NF_N_ and NF_N+1_) analysis in *geNorm* highlighted the need of at least two different genes for most of the conditions tested. The exception was the body parts comparison experiment in *C*. *littoralis*, for which no value was obtained below the V_n/n+1_ 0.15 cut-off, even when the number of genes considered for the analysis was increased. For that case, according to the *geNorm* manual, the recommended number of genes for normalization should be three, as has previously considered by other authors such as Lu *et al*.^47^ or Koramutla *et al*.^19^. On the other hand, Ling & Salvaterra^50^ pointed that the optimal number of reference genes should be given according to the minimal V_n/n+1_ value, which in this case also corresponds to three genes.

All reference genes selected after these analyses were tested in an actual qPCR experiment to complete their validation. A defensin gene was tested in *C*. *littoralis* after an infection, which seemed to increase its expression as it was showed in a previous work^37^, being in accordance to other studies performed for defensin genes in insects^51,52^. On the other hand, the ubiquitin UBC9 was tested for the rest of the conditions in both species, showing significant differences in the body parts comparison, primarily in gonads, as was previously checked in a different work^38^ and in other insects such as *Drosophila*^53^, which confirmed the relation of this gene with cicindelids reproductive system.

On the whole, this work identifies nine candidate reference genes and validates their suitability as normalizers in expression studies in two different species of tiger beetles, and later used in a relative expression experiment with two target genes which showed variation in their expression. As has previously concluded in several studies, this work proves the absence of a single and universal reference gene for both species and all conditions tested, and gene validations must be performed for every species and experiment before any expression analysis. Based on the results obtained with all four algorithms, the recommended genes are displayed in Table 4. Nevertheless, some genes seem to present a better stability across samples and species, and in most of the cases all genes showed values within the acceptable range of stability, considering them suitable as well. That is the case of *NADH* and, to a lesser extent, *ArgKin*, *Vatpase* and *B-t*. Other genes were top-ranked for some specific conditions but were discarded for other, such as *RPS18* or *Myo-Alc* for gonad development and sex comparison, respectively. This work is the first reference gene validation in cicindelids, an ecologically important group of predaceous beetles, and provides useful information to perform future genomic studies in other coleopteran species, either based on the concrete results of selected genes or on the procedures to carry out a proper validation to perform reliable expression studies.

**Table 4:** Selection of recommended genes for each species and condition according to the results of *geNorm*, *NormFinder*, *Bestkeeper* and *ΔC*_*T*_ algorithms.

Condition	Recommended genes	
	*C*. *campestris*	*C*. *littoralis*
Sex	B-t, Vatpase	Myo, Myo-Alc
Gonad development	RPS18, NADH	—
Infection	—	NADH, Myo
Body parts	Vatpase, RPS11	NADH, 60Sa, ArgKin

## Methods

### Insects

All individuals used in this study were captured directly from field, and subsequently treated, harvested and stored for experimental analyses. *C*. *campestris* imagoes were collected from Laguna del Arquillo (Albacete, Spain) while *C*. *littoralis* were collected from Laguna de Pétrola (Albacete, Spain).

### Total RNA extraction and cDNA synthesis

Three different conditions were tested for *C*. *campestris:* (i) male and female beetle comparisons; (ii) four different body parts including head, thorax, abdomen and gonads; (iii) developmental stage of their gonads, discerned based on the description reported by Paarmann^54^ and previously used by Rodríguez-García *et al*.^38^. The first two conditions were also tested for *C*. *littoralis*, with an additional new condition: infection of beetles with 1 μl of 1 mg/ml lipopolysaccharides (LPS) of *Escherichia coli* as previously performed by Rodríguez-García *et al*.^37^ and harvested after 12 hours of infection. Control samples were injected with 1 μl of insect saline solution. Each condition was replicated three times and each replicate was pooled with 3 different individual or body parts.

Insects and tissues dissected were stored in RNAlater (Qiagen, Crawley, UK) at −20 °C until RNA isolation. Total RNA from samples was extracted using TRIzol reagent (Life Technologies) following the manufacturer’s protocol, and isolated RNA was treated using a TURBO DNA-free Kit (Ambion, Life Technologies) to remove DNA contamination. RNA presence and quality was checked on a 1.5% agarose gel and quantified by NanoDrop 1000 from Thermo Fisher Scientific® (Waltham, MA, USA). First strand cDNA was obtained using PrimeScript^TM^ RT Reagent Kit (Takara Bio Inc., Japan) and store at −20 °C.

### ESTs analysis and selection of candidate HKGs

ESTs libraries from *C*. *campestris*^55^, *C*. *littoralis*^36^ and other unpublished sequences from *C*. *littoralis* were analysed in order to identify putative HKGs. Blast searches and contig annotations performed with Blast2go v2.5.0 software^56^ revealed nine genes known to function as housekeeping genes in other insect species, all of which were selected for further analyses.

### Real-time quantitative PCR

Primers for reverse transcription quantitative PCR (RT-qPCR) analyses designed with Primer Express 3 software (Applied Biosystems®) are shown in Table 1. A RT-PCR was performed and checked in an agarose gel to prove the single amplification and the predicted length of each gene (95 °C for 2 min; 40 cycles at 95 °C for 15 s, 60 °C for 30 s and 72 °C for 1 min; 72 °C for 5 min). Gene amplification efficiencies were calculated for all genes and both species using a serial dilution pool of cDNA with the quantification standard curve method, repeated in triplicate and carried out on a StepOnePlus™ Real-Time PCR System using SYBR® Green (Applied Biosystems®). Those genes with good efficiencies were used for RT-qPCR experiments on the same equipment with 1.5 µl of the same starting concentration of cDNA for each sample. All PCR conditions were as follows: one cycle at 95 °C for 2 min and 40 cycles at 95 °C for 15 s and 60 °C for 30 s. Experiments were performed with three biological and three technical replicates. Non template controls were used as negative control and melting curves analysis were carried out to assure the specificity of the amplifications.

Additionally, two target genes were amplified using the same instrument and conditions to perform a relative expression analysis using the reference genes previously selected for *C*. *campestris* (sex: B-t and Vatpase; gonad development: RPS18 and NADH; body parts: Vatpase and RPS11) and *C*. *littoralis* (sex: Myo and Myo-Alc; infection: NADH and B-t; body parts: NADH, 60Sa and ArgKin). Both target genes were amplified as described in previous works with previously described primers^37,38^. UBC9, an ubiquitin gene related to reproduction and sexual activity, was tested for all conditions and species with the exception of the infection in *C*. *littoralis*, for which a member of the defensin family (Clit-Def) was used to check changes in the expression.

### Data analysis

The raw C_T_ data were obtained with 7500 software v2.0.5 (Applied Biosystems®), and gene expression and stability were later analysed using four different tools and algorithms: *geNorm*^39^, *NormFinder*^40^, *BestKeeper*^41^ and *ΔC*_*T*_ *method*^42^.

*geNorm* calculates the average expression stability values (M) as the average pairwise of variation of one of the genes with all control genes present in the experiment. The most stable genes are those with the lowest M values. At the same time, it provides the optimal number of reference genes needed for normalization in expression measurements studies. *NormFinder* algorithm estimates the overall expression variation of the candidate reference genes and also the variation between sample subgroups of the same set. The stability value provides information about the reference genes, with lower values meaning higher stability, as occurs with *geNorm* algorithm. Both *geNorm* and *NormFinder* use quantities transformed to a linear scale as input. Similarly, the main principle of *Bestkeeper* is that reference genes should present the same expression patterns across samples. Thus, this software uses raw C_T_ values to determine the best correlated reference genes and combines them into an index. The lower the standard deviation values obtained are the more stability present the reference genes tested. Finally, *ΔC*_*T*_ *method* also uses raw C_T_ values and compares them between pairs of genes within each sample to rank them considering the standard deviation mean for each reference gene.

For qPCR analyses with both target genes, gene expression was obtained considering all genes efficiencies as described by Pfaffl^57^. To obtain the statistical significance, a Student’s t-test was performed for sex comparison, gonad development and infection analyses and a one-way ANOVA with a Tukey’s HSD post-hoc test for body parts comparison with the software GraphPad Prism version 5.00 (GraphPad Software, San Diego California USA, www.graphpad.com).
